# Modulation of anxiety by cortical serotonin 1A receptors

**DOI:** 10.3389/fnbeh.2015.00048

**Published:** 2015-02-24

**Authors:** Lukasz Piszczek, Agnieszka Piszczek, Joanna Kuczmanska, Enrica Audero, Cornelius T. Gross

**Affiliations:** Mouse Biology Unit, European Molecular Biology LaboratoryMonterotondo, Italy

**Keywords:** serotonin, cortex, anxiety, serotonin 1A receptor, mouse model

## Abstract

Serotonin (5-HT) plays an important role in the modulation of behavior across animal species. The serotonin 1A receptor (Htr1a) is an inhibitory G-protein coupled receptor that is expressed both on serotonin and non-serotonin neurons in mammals. Mice lacking Htr1a show increased anxiety behavior suggesting that its activation by serotonin has an anxiolytic effect. This outcome can be mediated by either Htr1a population present on serotonin (auto-receptor) or non-serotonin neurons (hetero-receptor), or both. In addition, both transgenic and pharmacological studies have shown that serotonin acts on Htr1a during development to modulate anxiety in adulthood, demonstrating a function for this receptor in the maturation of anxiety circuits in the brain. However, previous studies have been equivocal about which Htr1a population modulates anxiety behavior, with some studies showing a role of Htr1a hetero-receptor and others implicating the auto-receptor. In particular, cell-type specific rescue and suppression of Htr1a expression in either forebrain principal neurons or brainstem serotonin neurons reached opposite conclusions about the role of the two populations in the anxiety phenotype of the knockout. One interpretation of these apparently contradictory findings is that the modulating role of these two populations depends on each other. Here we use a novel Cre-dependent inducible allele of *Htr1a* in mice to show that expression of Htr1a in cortical principal neurons is sufficient to modulate anxiety. Together with previous findings, these results support a hetero/auto-receptor interaction model for Htr1a function in anxiety.

## Introduction

The major inhibitory serotonin (5-HT) receptor, serotonin 1A receptor (Htr1a), is found in the brain in two distinct populations, autoreceptors and heteroreceptors. Auto-receptors are expressed on serotonin neurons in the raphe nuclei and provide a regulatory feedback loop by inhibiting the firing rate of serotonin cells (Pompeiano et al., 1992; Blier et al., 1998; Evrard et al., 1999). Heteroreceptors are expressed widely in the brain, including cortex, amygdala and hippocampus, on non-serotonin neurons (Pompeiano et al., 1992; Casanovas et al., 1999). Mice lacking the *Htr1a* gene show increased anxiety and stress responses in a number of behavioral tests (Ramboz et al., 1998; Bonasera and Tecott, 2000; Ase et al., 2002; Toth, 2003). On the other hand, pharmacological activation of *Htr1a* receptors reduces anxiety (File and Gonzalez, 1996) and Htr1a agonists have been used successfully in the clinic to treat anxiety disorders (Artigas et al., 1996; Lader and Scotto, 1998; Trivedi et al., 2006). In humans, positron emission tomography studies show correlations between trait anxiety and lower HTR1A binding (Albert, 2012). Likewise such correlations have also been seen for other mental traits (Meltzer et al., 2004; Neumeister et al., 2004; Spindelegger et al., 2009; Shrestha et al., 2012). Moreover, a common genetic variant in the promoter of HTR1A in humans, that is thought to reduce its expression, is associated with suicide and depression (Lemonde et al., 2003; Parsey et al., 2006). Findings from tissue-specific injections of Htr1a agonists in rodents suggest that different populations of Htr1a may have opposing roles in anxiety modulation, as agonists injected into the hippocampus or amygdala are anxiogenic, while injections into the raphe are anxiolytic (File et al., 1996; Gonzalez et al., 1996; Akimova et al., 2009).

Cell-type specific manipulation of Htr1a in transgenic mice has been a powerful approach to examine the role Htr1a in anxiety and to define the cell-types and circuits involved. Previous studies testing the role of Htr1a hetero-receptors on anxiety either used expression of Htr1a under control of the *Camk2a* promoter on an otherwise knockout background (Gross et al., 2002) or used the suppression of endogenous Htr1a in Camk2a-expressing cells (Richardson-Jones et al., 2011). The first study showed that rescue of Htr1a expression was able to reverse the anxiety phenotype of the knockout, while in the latter no effect of suppression of Htr1a expression on anxiety behavior was seen. However, concerns raised in the first investigation by the ectopic expression of Htr1a (e.g., in striatal principal neurons) rendered direct comparison of both studies difficult. Here we developed a new conditional rescue allele of *Htr1a* and used it to test the role of Htr1a hetero-receptors in anxiety. We show that increasing Htr1a3 expression selectively in cortical principal neurons is associated with decreasing anxiety in mice. These data demonstrate that Htr1a impacts anxiety at least in part by modulating cortical function.

## Materials and methods

### Gene targeting

Genomic sequence harboring the *Htr1a* coding sequence and 5′UTR was amplified using tail genomic DNA from C57BL/6N mice and cloned into a pcDNA3 expression plasmid (Invitrogen, Carlsbad, CA, USA). The tdTomato coding sequence (Silva et al., 2013) was amplified from pRSET-B-tdTomato (kindly provided by Roger Tsien, University of California, San Diego, USA) and cloned downstream of the Htr1a open reading frame separated by a P2A sequence (Holst et al., 2006). The pBS-XZ/keo/STOP/tetO cassette (kindly provided by Xiao Xi Zhuang, University of Chicago, USA) was subcloned upstream of the Htr1a open reading frame and 5′ and 3′ homology arms were introduced flanking the construct. A genomic fragment containing the *Htr1a* locus was retrieved from a 220 kb mouse BAC (RP23-146M15, Chori-BACPAC Resources, Oakland, USA) using recombineering. The entire STOP-Htr1a-P2A-tdTomato cassette was then recombined into the retrieved sequence and the resulting construct used to target mouse embryonic stem cells.

### cAMP assay

Chinese hamster ovary (CHO) cells (2.5 × 10^6^) were seeded on a 10 mm diameter dish. After 24 h cells were transfected according to the manufacturer's instructions (FuGENE HD; cat. no. 0470969100; Roche Diagnostics GmbH, Mannheim, Germany) with (1) pmaxGFP (kindly provided by Paul Heppenstall, EMBL), (2) pcDNA3-Htr1a-P2A-tdTomato, or (3) a mixture of pmaxGFP and pcDNA3-Htr1a. Forty 8 h later cells were harvested by trypsinization, resuspended in single cell suspension and sorted by FACS using GFP (1 and 3) or tdTomato (2) channels. Fifteen thousand cells were seeded per well in a 96-well plate. Eighteen hours later cAMP assay was performed using cAMP-Glo kit according to manufacturer instruction (Promega Madison, USA).

### Western blotting

CHO cells were seeded and transfected 24 h afterwards with either empty pcDNA3 vector, pcDNA3-Htr1a-P2A-tdTomato or pcDNA3-Htr1a-P2A^*^-tdTomato, according to manufacturer instructions (FuGENE HD; Roche Diagnostics, Mannheim, Germany). Cells were collected by trypsinization, centrifuged and washed twice with ice cold PBS and resuspended in hot 4x Laemmli buffer (0.22 M Tris-HCl, pH 6.8; 4.4% SDS; 5% β-mercaptoethanol; 44% glycerol; 0.05% bromophenol blue), boiled at 95°C for 10 min and passed at least 5 times through 1 ml syringe. The protein extract was stored at -80°C until used. Western blotting was performed as described previously (Lo Iacono and Gross, 2008) using 1:1000 Living Colors® DsRed (Takara Bio Europe/Clontech, Saint-Germain-en-Laye, France) primary antibody for detection of tdTomato and 1:4000 secondary HRP-conjugated rabbit IgG antibody (NA934 Amersham Biosciences, Otelfingen, Switzerland). The blots were then re-analyzed using 1:5000 α-actin primary antibody (Millipore, Billerica, USA) and HRP-coniugated mouse IgG antibody (NA931 Amersham Biosciences, Otelfingen, Switzerland).

### Htr1a^cR^ mouse line generation

The Htr1a targeting construct was electroporated into A9 ES cells (Nakashima et al., 2011; C57BL/6x129/Sv) kindly provided by Donal O'Carroll (EMBL) and Anton Wutz (ETH, Switzerland). Southern blot was used to detect proper integration of the construct. Positive clones were injected into C57BL/6 8-cell-stage embryos for generation of fully ES-cell-derived mice as described elsewhere (De Fazio et al., 2011). ES-cell-derived mice were bred with C57BL/6 mice for >5 generations to establish the *Htr1a*^cR^ line. To obtain our behavioral cohorts, both Emx1-Cre (*Emx*1^Cre/+^,Iwasato et al., 2000, 2004; >6 generations C57BL/6) and Htr1a-cR (*Htr1a*^cR/+^) lines were crossed with the constitutive Htr1a knockout line (*Htr1a*^*KO/KO*^; Ramboz et al., 1998; congenic C57BL/6, Lo Iacono and Gross, 2008). The offspring were intercrossed (*Emx*1^Cre/+^; *Htr1a*^KO/+^ × *Htr1a*^cR/KO^) to produce the parents (*Emx*1^Cre/+^; *Htr1a*^cR/KO^ × *Htr1a*^cR/KO^) of the behavioral cohorts: (1) *Emx*1^Cre/+^; *Htr1a*^cR/cR^, (2) *Emx*1^+/+^; *Htr1a*^cR/cR^, (3)*Emx*1^Cre/+^; *Htr1a*^KO/KO^, and (4) *Emx*1^+/+^; *Htr1a*^KO/KO^. The genetic background of the mice analyzed in the behavioral part of the study were thus >6 generations backcrossed to C57BL/6. Care was taken to exclude transgenic animals (36% of Cre+, cKO+ mice) in which Cre-mediated deletion of the cKO cassette was detected in blood and that presumably had undergone widespread deletion (Iwasato et al., 2004; Zeller et al., 2008). Such deletion was seen predominantly in offspring in which the transgenes were inherited via the father, and may be due to activity of Emx1-Cre in the male germline. Breedings between heterozygous *Htr1a* knockout mice that were siblings of the animals described above were carried out in parallel to obtain wild-type and knockout littermates for gene expression and behavioral testing.

### Other mouse lines used in the study

The Emx1-Cre line (Iwasato et al., 2000, 2004), kindly provided by Dr. Takuji Iwasato, was crossed to a FLP-deleter strain (Farley et al., 2000; congenic C57BL/6) in order to excise the Neo cassette, which has been reported to drive ectopic expression (Iwasato et al., 2004). The line was then further backcrossed to C57BL/6 for >5 generations. The HPRT Cre-deleter line (Tang et al., 2002), kindly provided by Francois Spitz (EMBL), and *Slc*6*a*4^*mtTA*/+^ animals (Audero et al., 2008), kindly provided by Enrica Audero, were maintained on a mixed 129S6/SvEvTac;C57BL/6;CBA background.

### Animal husbandry

Animals were housed in groups of two to four per cage with *ad libitum* access to food and water. Animals were maintained on a 12:12 light/dark schedule (lights on at 7:00, off at 19:00). All testing was conducted during the light period. All experiments followed protocols approved by the Italian Ministry of Health and were carried out in accordance with the European Communities Council Directive of 24 November 1986 (86/609/EEC).

### Radioligand binding

Mice were sacrificed by cervical dislocation and decapitation. Extracted brains were frozen immediately on dry ice and kept at -80°C until sectioning. Tissue was cryosectioned at 18 μm and thaw-mounted on glass slides (Superfrost, Fisher Scientific, Pittsburgh, USA). The slides were kept at -80°C until further processing. Receptor expression was assessed by incubation with 4-(2-methoxyphenyl)-1-[2-(n-2″-pyridinyl)-p-[^125^I]iodobenzamido]ethylpiperazine (^125^I-MPPI, Perkin-Elmer, Waltham, USA) washing briefly with water followed by exposure to a film and quantitative autoradiography as previously described (Piszczek et al., 2013). For validating expression by Cre-deleter mediated excision two to four animals per group (*Htr1a*^cR/cR^, *Htr1a*^cR/cR^;*Hprt*^Cre/+^,*Htr1a*^+/+^, and *Htr1a*^KO/KO^) and 3–12 sections per animal were quantified and averaged. For validating expression following Emx1-Cre mediated excision five to eight animals per group (*Htr1a*^cR/cR^, *Htr1a*^cR/cR^;*Emx*1^Cre/+^, *Htr1a*^+/+^, and *Htr1a*^KO/KO^) and 3–12 sections per animal were quantified and averaged.

### Immunofluorescence

Mice were anesthetized intraperitoneally with Avertin (Sigma-Aldrich, St Louis, USA) and perfused transcardially with 4% paraformaldehyde. Brains were postfixed overnight at 4°C and sectioned on a vibratome (70 μm; Leica Microsystems, Wetzlar, Germany). Free floating sections were stained with primary antibodies overnight at 4°C (1:1000 mouse α-GAD67, Millipore, Billerica, USA; 1:1000 rabbit α-RFP, Rockland Immunochemicals Inc., Gilbertsville, USA) and incubated with secondary antibodies (2 h at room temperature; mouse IgG A488 or rabbit IgG A594, Molecular Probes/Invitrogen, Carlsbad, USA). Confocal microscopy was performed with a TCS-SP5 (Leica) Laser Scanning System. The images were processed using the ImageJ software (NIH, Bethesda, MD).

### Behavioral testing

Mice were tested at 8–12 weeks of age, unless otherwise indicated. For all tests both males and females were used. Behavioral tests were separated by 1 week intervals to reduce inter-test interactions. The open field test was carried out as previously described (Lo Iacono and Gross, 2008; Piszczek et al., 2013) and lasted 45 min. Dependent measures were: total distance traveled, percentage of distance in the center (distance traveled in the center divided by the total distance traveled), and time spent in the center and were scored automatically by a videotracking system (TSE Systems, Bad Homburg, Germany). The elevated plus maze test was carried out as previously described (Piszczek et al., 2013) and lasted 5 min. Initially, mice were placed in the central platform facing a closed arm and behavioral measures (time spent in open arms, time spent in closed arms, percentage of distance traveled in open arms, and total distance traveled) were scored using a videotracking system (TSE Systems) or scored manually (head dips). The light/dark test was performed in a similar manner as described elsewhere (Klemenhagen et al., 2006). For this test the same apparatus as for the open field was used, with the following modifications: to each open field apparatus a black, opaque box was inserted with height and width allowing tight insertion and having half the length of the open field box, with an opening in the middle of the box to allow mouse access. The setup was illuminated indirectly with two 500 W white lamps, resulting in 150-170 lux in the light compartment. The test lasted 20 min. Dependent measures were: distance traveled in light compartment, time spent in the light compartment, number of transitions between light and dark compartments and were scored automatically by a videotracking system (TSE Systems). The tail suspension test was carried out as previously described (Carola et al., 2008). To avoid tail climbing that is frequent in female mice a short section of plastic tubing (cut from 15 ml tube) was placed over the tail of all animals. Periods of immobility were scored manually for 6 min.

### Statistical analysis

In general, analysis of behavioral and physiological responses was performed using *Prism 4* software (GraphPad Software, San Diego, USA). The effect of genotype was assessed by ANOVA using repeated measures when appropriate or Student *t-test*. In case of significance ANOVA was followed by Tukey's multiple comparison *post hoc* test to compare individual genotypes. The effect of 8-OH-DPAT on cAMP levels in cells was analyzed by Two-Way ANOVA and followed in cases of significance by Bonferroni *post hoc* test.

## Results

### Design of Cre and tTA-inducible *Htr1a* allele

**Figure 1:**
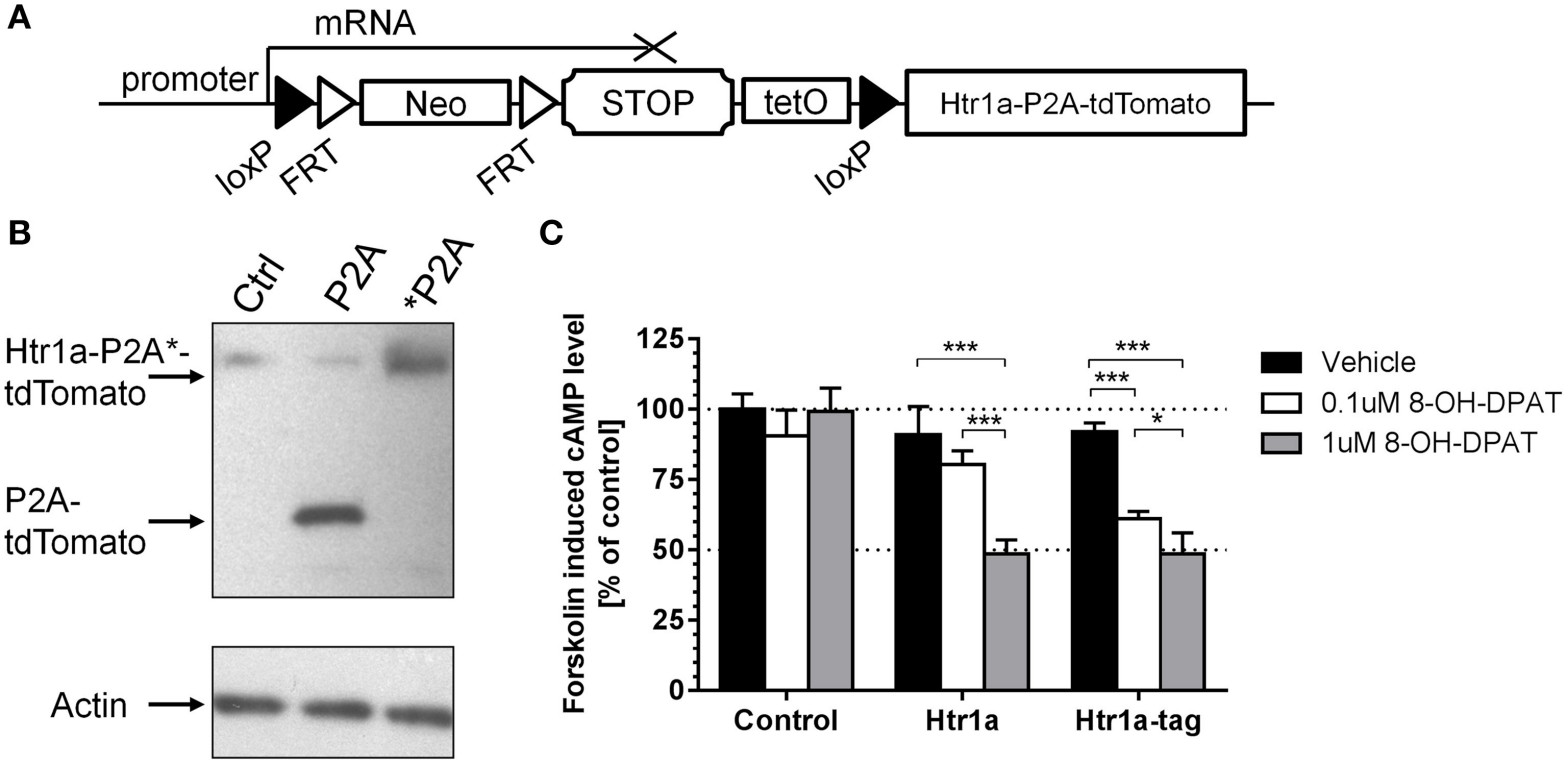
**Cre/tTA-inducible and Tomato-tagged allele of *Htr1a*. (A)** An overview of the *Htr1a*^cR^ mouse line showing the loxP-flanked transcriptional stop cassette targeted to the 5′ UTR of *Htr1a* and the tdTomato reporter fused to the C-terminus of Htr1a via the self-cleaving P2A peptide. **(B)** Western blot probed with anti-RFP or anti-Actin control antibodies of extracts from CHO cells transfected with Htr1a-P2A-tdTomato or uncleavable Htr1a-^*^P2A-tdTomato plasmid revealed the expected cleaved P2A-tdTomato band and uncleaved Htr1a-^*^P2A-tdTomato band, respectively. **(C)** Quantification of forskolin-induced cAMP levels in CHO cells transfected with Htr1a or Htr1a-P2A-tdTomato plasmid and treated with low or high dose of the selective Htr1a agonist 8-OH-DPAT revealed a similar dose-dependent blockade by agonist treated wild-type or tagged Htr1a (*N* = 4–5, mean ± SD, ^*^P < 0.05, ^***^P < 0.001).

To create a Cre-inducible *Htr1a* allele (Figure 1A; cR; *Htr1a^cR^*) a loxP-flanked transcriptional STOP cassette (His3-SV40pA sequence; Lakso et al., 1992; Sauer, 1993) was inserted into the 5′UTR of the *Htr1a* gene in embryonic stem cells *via* gene targeting. To allow tTA-dependent transactivation of Htr1a from the same allele, a tetracycline operator (7xtetO) was placed upstream of the STOP cassette (Lewandoski, 2001). To fluorescently tag cells expressing Htr1a, a tdTomato protein was fused to the C-terminus of Htr1a via a self-cleaving P2A sequence (Shaner et al., 2004; Szymczak et al., 2004; Holst et al., 2006; Shuen et al., 2008).

### *In vitro* testing of Htr1a-P2A-tdTomato functionality

The P2A sequence has been successfully used before in both *in vitro* and *in vivo* experiments for multi-cistronic expression of proteins, resulting in their post-translational separation (Szymczak et al., 2004; Holst et al., 2006). However, this feature was first tested with our *Htr1a* constructs. CHO cells were transfected with expression vectors carrying either a Htr1a-P2A-tdTomato (P2A) construct or a modified version in which the P2A sequence had been mutated to a non-cleavable form (^*^P2A; Szymczak et al., 2004). The cleavage was then assessed by Western Blot using an anti-dsRed antibody that recognizes tdTomato. As expected, the translation product of the cleavable form has migrated faster than its non-cleavable counterpart, indicating its lower molecular weight (Figure 1B). This result suggests separation of the tdTomato from Htr1a *in vitro*.

Htr1a receptors are negatively coupled to adenylyl cyclase and their activation results in reduction of intracellular cAMP levels (Fargin et al., 1989). The 18 amino acid peptide left on the C-terminus of Htr1a protein by P2A cleavage could possibly impair receptor functionality. To test this possibility the wild-type Htr1a receptor or Htr1a-P2A-tdTomato was expressed in CHO cells and the change in forskolin-induced cAMP levels upon exposure to the Htr1a agonist 8-OH-DPAT (Figure 1C) was measured. Two-Way ANOVA (treatment-by-group) revealed a significant main effect of treatment [*F*_(2, 30)_ = 64.83, *P* < 0.0001], group [*F*_(2, 30)_ = 72.99, *P* < 0.0001], and treatment-by-group interaction [*F*_(4, 30)_ = 19.95, *P* < 0.0001]. There was no statistically significant effect for vehicle treatment between groups (*post-hoc*, *P* > 0.05) and no effect of drug on the cells transfected with empty vector (*post-hoc*, *P* > 0.05).

### Creation and validation of H*tr1a^cR^* allele

**Figure 2:**
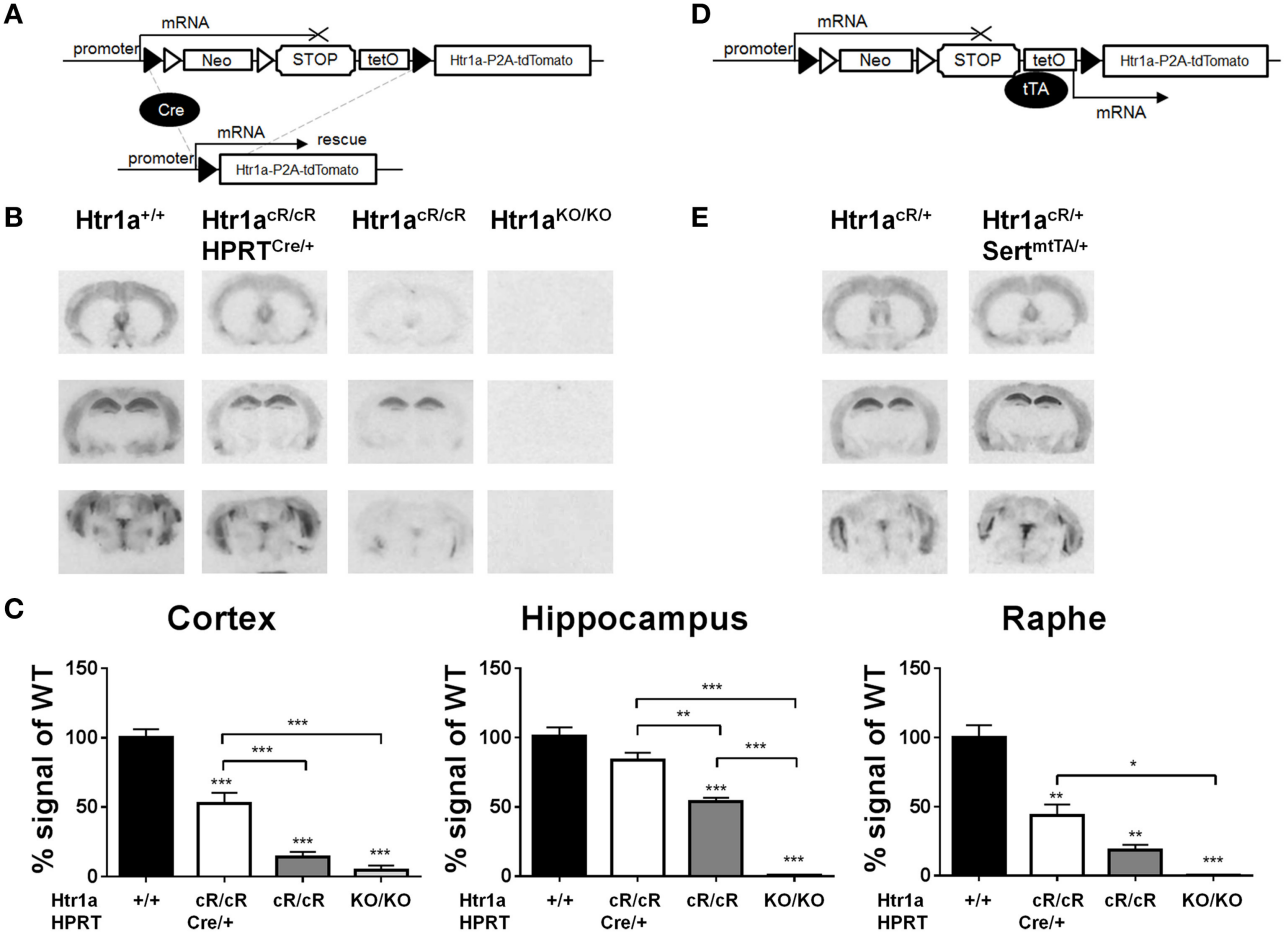
**Cre- and tTA-inducible expression of Htr1a. (A)** Cre-mediated excision of the STOP cassette in *Htr1a*^cR^ mice allows expression of Htr1a from its native promoter. **(B)** Representative autoradiographs of ^125^I-MPPI binding to coronal mouse brain sections containing septum (top), hippocampus (middle), and raphe (bottom) from adult wild-type (*Htr1a*^+/+^), knockout (*Htr1a*^KO/KO^), conditional rescue (*Htr1a*^cR/cR^), and constitutive rescue (*Htr1a*^cR/cR^; *Hprt*^Cre/+^) mice. **(C)** Quantification of normalized ^125^I-MPPI signal in auditory cortex (Cortex), CA1 hippocampus (Hippocampus) and dorsal raphe (Raphe) confirmed increased expression of Htr1a in the constitutive rescue as compared to the conditional rescue line (*N* = 2 − 4; mean ± SEM, ^*^P < 0.05, ^**^P < 0.01, ^***^P < 0.001; asterisks above bars indicate *P*-value vs. wild-type). **(D)** tTA-mediated activation of transcription in *Htr1a*^cR^ mice allows expression of Htr1a from a heterologous promoter. **(E)** Representative autoradiographs of ^125^I-MPPI binding to coronal mouse brain sections containing septum, hippocampus, and raphe nuclei from adult animals expressing tTA in serotonin neurons (*Htr1a*^cR/+^; *Slc6a4*^*mtTA*/+^) revealed increased Htr1a expression in raphe nuclei, but not septum, cortex, or hippocampus when compared to control littermates (*Htr1a*^cR/+^; *Slc*6*a*4^+/+^).

Next, murine ES cells were targeted with the targeting construct shown in Figure 1A, selected positive clones, and piezo-drill assisted morula injection was used to generate mice carrying the Cre/tTA-conditional *Htr1a*^cR^ allele. *Htr1a*^cR/cR^ mice were then crossed to the *Hprt*-Cre line (Tang et al., 2002) to remove the loxP-flanked STOP cassette. Autoradiography was then used to quantify Htr1a protein expression in tissue sections of the resulting ubiquitous rescue mice (*Htr1a*^cR/cR^; *Hprt*^Cre/+^) and non-Cre carrying littermates (*Htr1a*^cR/cR^) (Figure 2A). Excision of the STOP cassette resulted in Htr1a protein expression in a pattern indistinguishable from that seen in wild-type mice. Interestingly, Htr1a expression in *Htr1a*^cR/cR^; *Hprt*^Cre/+^ mice was lower than that seen in wild-type animals suggesting that the remaining exogenous sequences in the *Htr1a* locus (e.g., loxP, P2A-tdTomato, etc.) impeded full expression of Htr1a. Moreover, Htr1a protein was also detected at low levels in the non-Cre carrying *Htr1a*^cR/cR^ littermates indicating that the STOP cassette did not completely block Htr1a transcription (Figure 2B). Importantly, the expression pattern in the *Htr1a*^cR/cR^ animals was indistinguishable from that seen in wild-type mice, suggesting that it was driven by endogenous *Htr1a* regulatory sequences. Nevertheless, quantification of Htr1a binding confirmed a significant effect of genotype in cortex [ANOVA, main effect of genotype: *F*_(3, 11)_ = 58.14, *P* < 0.0001], hippocampus [ANOVA, main effect of genotype: *F*_(3, 10)_ = 56.13, *P* < 0.0001], and dorsal raphe [ANOVA, main effect of genotype: *F*_(3, 8)_ = 25.37, *P* = 0.0002]. In addition, the *Htr1a*^cR/cR^ line was crossed to a Flp-deleter transgenic line (Farley et al., 2000) to investigate whether the FRT-flanked neomycin selection cassette was responsible for the leakiness of the STOP cassette. Expression analysis demonstrated that deletion of the neomycin gene cassette resulted in higher rather than lower tdTomato and Htr1a expression (data not shown) arguing against a contribution of the neomycin gene to leakiness. Finally, the *Htr1a*^cR^ line was crossed with the *Slc6a4*-tTA line (Audero et al., 2008) to assess tTA-inducible expression of Htr1a (Figure 2D). As expected, expression of Htr1a was significantly induced in raphe nuclei (Figure 2E).

### Cell-type specific Htr1a expression in Htr1aR mice

**Figure 3:**
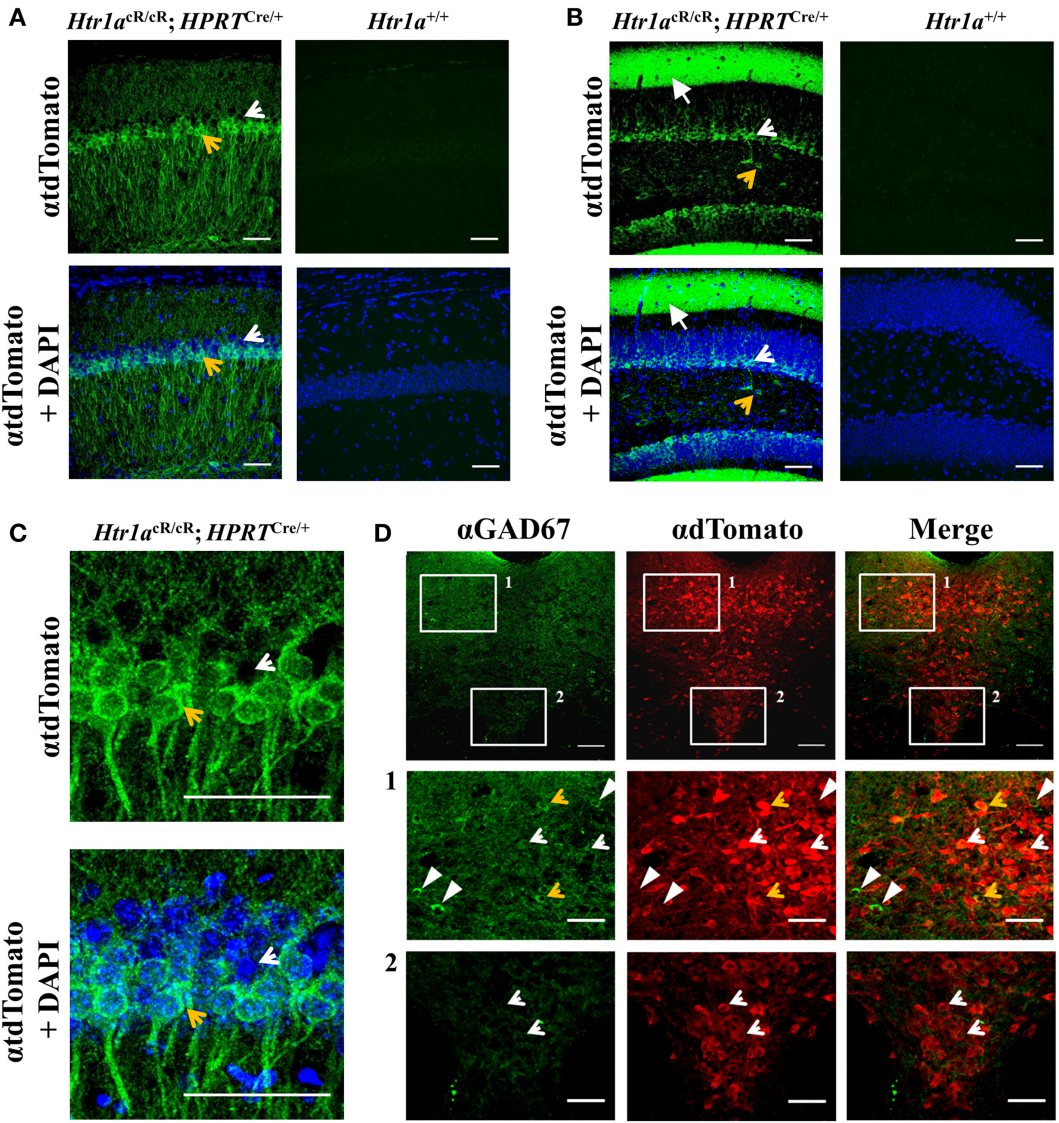
**Cell-type specific gene expression in *Htr1a*R mice**. Immunohistochemistry revealed tdTomato reporter protein expression in **(A)** CA1 pyramidal cells of the hippocampus and **(B)** granule cells and processes of the outer molecular layer of the dentate gyrus in *Htr1a*R (*Htr1a*^cR/cR^; *HPRT*^Cre/+^), but not wild-type mice. Note the preferential expression in the ventral portion of the CA1 pyramidal layer (tdTomato positive cell indicated by an yellow arrow; scale bar = 50 μm; higher magnification shown in **(C)** and in scattered cells in the hilus of the dentate gyrus (tdTomato negative cell indicated by an white arrow; scale bar = 50 μm). Overall anatomy is highlighted by DAPI counterstain (blue) in lower panels. **(D)** Double-immunofluorescence for the GABAergic marker Gad67 and tdTomato reporter protein revealed co-labeled cells in the lateral wings, but not midline of the dorsal raphe nucleus (orange arrows; scale bar = top 100 μm, middle and lower = 40 μm) Single labeled cells are marked as white arrowheads (Gad76) or white arrows (tdTomato).

Consistent with expression of Htr1a restricted to principal cells in cortical structures, tdTomato expression in the hippocampus of ubiquitous rescue mice (named Htr1aR, *Htr1a*^cR/cR^; *Hprt*^Cre/+^), was restricted to pyramidal and granule cells in the CA1 and dentate gyrus, respectively (Figures 3A,B). However, in the raphe nuclei Htr1a expression has been reported in both serotonergic and GABAergic neurons (Bonnavion et al., 2010) with the latter being suspected to provide inhibitory input to serotonin neurons (Wang et al., 1992; Gervasoni et al., 2000) and receive glutamatergic inputs from forebrain regions (Hajós et al., 1998, 2003, 1999; Boothman and Sharp, 2005). Consistent with the expression of Htr1a autoreceptors and heteroreceptors in the raphe, tdTomato expression in the analyzed mice was observed in both serotonergic and GABAergic neurons in the lateral wings of the dorsal raphe, while in the midline it was restricted to serotonergic neurons (Figure 3C).

### Cortex-specific increase of Htr1a expression is associated with decreased anxiety

**Figure 4:**
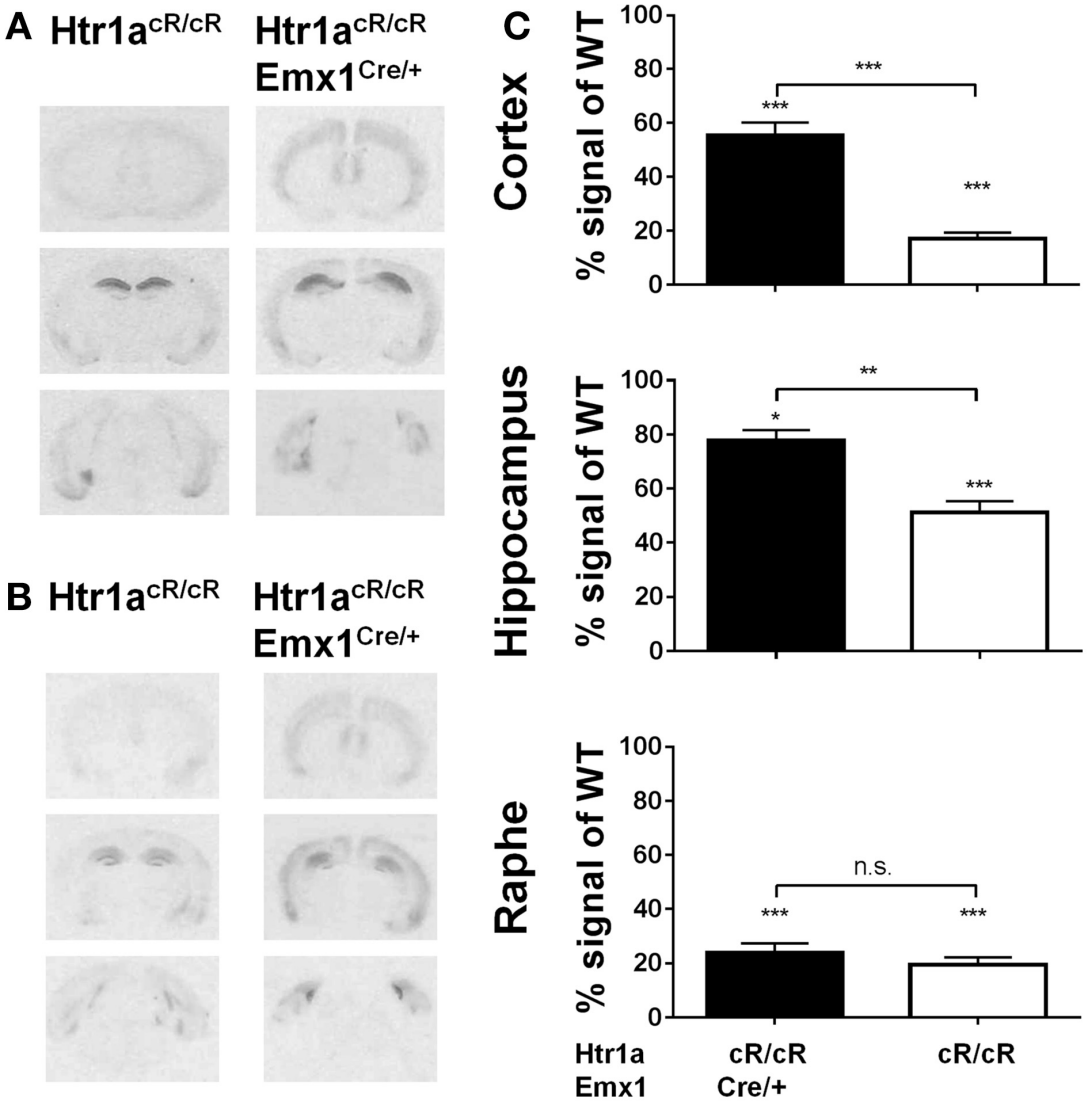
**Selective rescue of Htr1a expression in cortex. (A)** Representative autoradiographs of ^125^I-MPPI binding to coronal mouse brain sections containing septum, hippocampus, and raphe nuclei from control (*Htr1a*^cR/cR^) and adult cortex rescue (*Htr1a*^*cR/cR*^; *Emx*1^Cre/+^). **(B)** Representative autoradiographs of ^125^I-MPPI binding to coronal mouse brain sections containing septum, hippocampus, and raphe nuclei from control (*Htr1a*^cR/cR^) and cortical rescue (*Htr1a*^cR/cR^; *Emx*1^Cre/+^) mice at postnatal day 15. **(C)** Quantification of normalized ^125^I-MPPI signal in auditory cortex (Cortex), CA1 hippocampus (Hippocampus), and dorsal raphe (Raphe) confirmed tissue-specific rescue of Htr1a expression in cortex and hippocampus in adult animals (*N* = 5–8, mean ± SEM, ^*^P < 0.05, ^**^P < 0.01, ^***^P < 0.001; asterisks above bars indicate *P*-value vs. wild-type).

Our earlier data suggested that expression of Htr1a in forebrain principal neurons was sufficient to reverse the increased anxiety phenotype of *Htr1a* knockout mice (Gross et al., 2002). However, more recent data showed that suppression of Htr1a expression in forebrain principal neurons of wild-type mice did not alter anxiety behavior, and it has been argued that ectopic expression of Htr1a in the original study may have been responsible for the rescue phenotype and the apparent discrepancy between these findings (Richardson-Jones et al., 2011). An alternative possibility is that Htr1a expression in principal forebrain neurons is sufficient, but not necessary to modulate anxiety (Piszczek et al., 2013). To test this hypothesis *Htr1a*^cR/cR^ animals were crossed to *Emx1*-Cre transgenic mice (*Emx*1^Cre^; Iwasato et al., 2000) in order to increase Htr1a expression selectively in principal cortical neurons and examine whether expression of Htr1a under control of its endogenous promoter in this cell-type was sufficient to modulate anxiety behavior. As expected, double transgenic animals (*Htr1a*^cR/cR^; *Emx*1^Cre/+^) showed increased expression of Htr1a in cortex and hippocampus, but not in raphe nuclei when compared to non-Cre expressing littermates (Figure 4A). Increased expression was also observed in other cortical-related structures, such as basolateral amygdala. Importantly, no ectopic Htr1a expression was detected. Quantification of the signal (Figure 4C) showed a significant genotype effect on Htr1a expression in cortex [ANOVA, main effect of genotype: *F*_(3, 24)_ = 76.98, *P* < 0.0001], hippocampus [ANOVA, main effect of genotype: *F*_(3, 25)_ = 70.28, *P* < 0.0001], and dorsal raphe nucleus [ANOVA, main effect of genotype: *F*_(3, 20)_ = 45.79, *P* < 0.0001]. Finally, increased Htr1a expression in hippocampus and cortex was also seen at postnatal day 15 (Figure 4B), the critical period in which Htr1a is required for the modulation of the adult anxiety phenotype (Gross et al., 2002; Lo Iacono and Gross, 2008).

**Figure 5:**
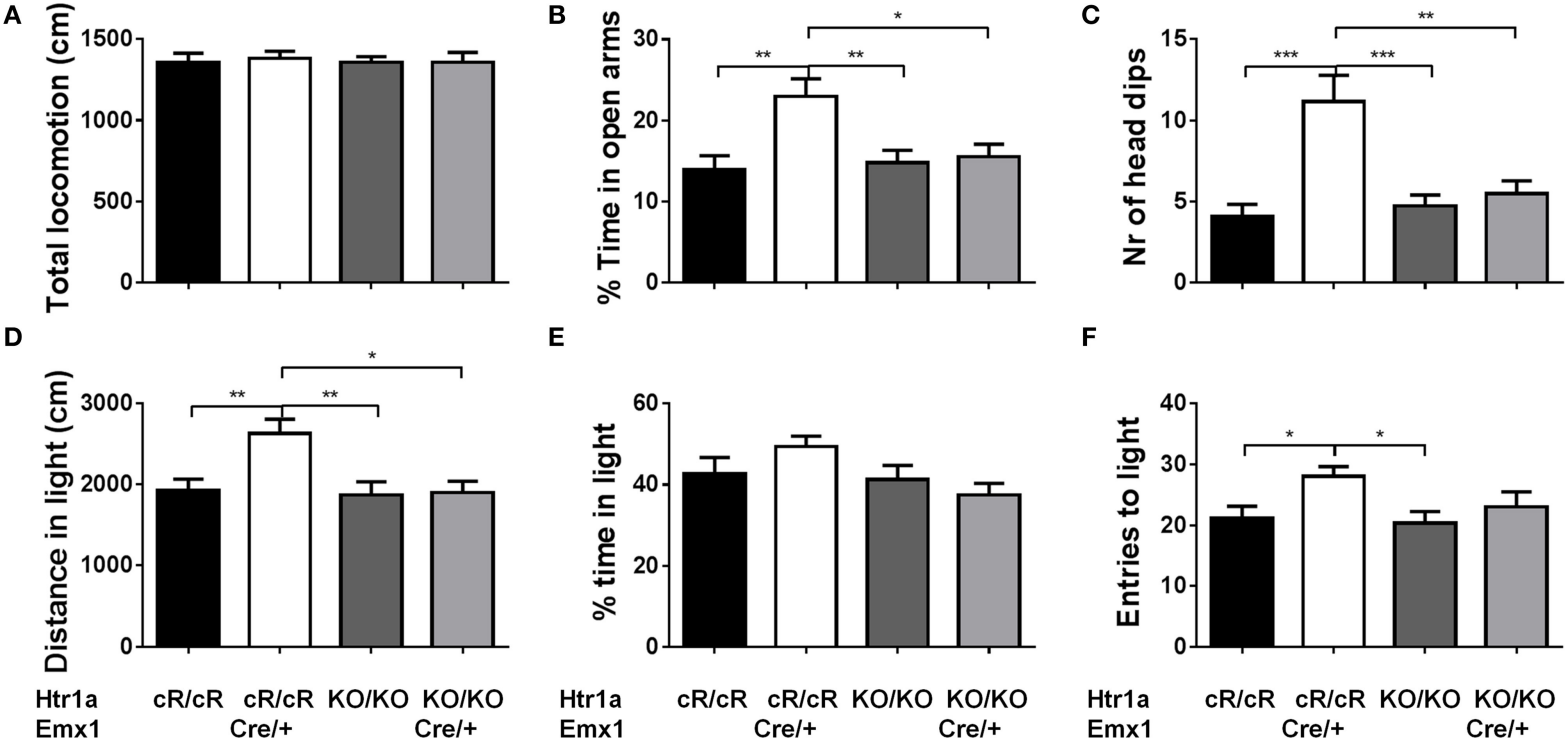
**Decreased anxiety in cortex rescue animals**. Testing of control (*Htr1a*^cR/cR^), cortical rescue (*Htr1a*^cR/cR^; *Emx*1^Cre/+^), knockout (*Htr1a*^KO/KO^), and knockout mice carrying the *Emx1*-Cre allele (*Htr1a*^KO/KO^; *Emx*1^Cre/+^) in the elevated plus (top row) and dark/light (bottom row) tests. No significant effect of genotype was seen on **(A)** total locomotion in the elevated plus maze. However, cortical rescue mice showed a significant increase in **(B)** time in the open arms and **(C)** head dips in the elevated-plus maze when compared to all other groups (*Htr1a*^cR/cR^, *N* = 27; *Htr1a*^cR/cR^; *Emx*1^Cre/+^, *N* = 25; *Htr1a*^KO/KO^, *N* = 35; *Htr1a*^KO/KO^; *Emx*1^Cre/+^, *N* = 25). Cortical rescue mice also showed a significant increase in **(D)** distance in the light compartment and a trend for an increase in **(E)** time in the light compartment and **(F)** entries into the light compartment in the dark/light test when compared to all other groups (*Htr1a*^cR/cR^, *N* = 25; *Htr1a*^cR/cR^; *Emx*1^Cre/+^, *N* = 25; *Htr1a*^KO/KO^, *N* = 22; *Htr1a*^KO/KO^; *Emx*1^Cre/+^, *N* = 18; mean ± SEM ^*^P < 0.05, ^**^P < 0.01, ^***^P < 0.001).

**Figure 6:**
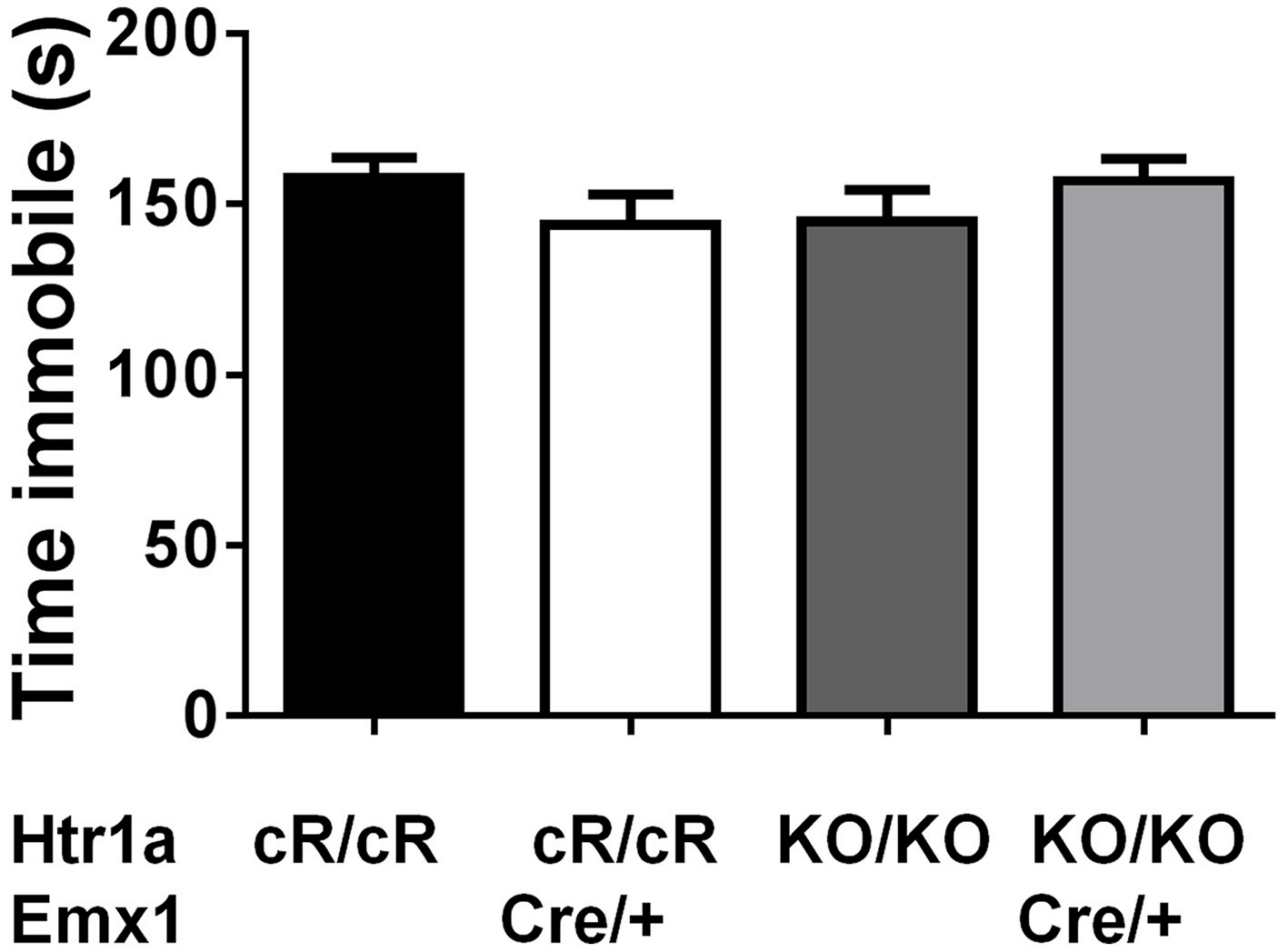
**No change in immobility in cortex rescue mice**. No significant effect of genotype was seen on immobility in the tail suspension test in control (*Htr1a*^cR/cR^, *N* = 27), cortical rescue (*Htr1a*^cR/cR^; *Emx*1^Cre/+^, *N* = 25), knockout (*Htr1a*^KO/KO^, *N* = 35), and knockout mice carrying the *Emx1*-Cre allele (*Htr1a*^KO/KO^; *Emx*1^Cre/+^, *N* = 25; mean ± SEM).

Next, anxiety behavior in adult cortical rescue (*Htr1a*^cR/cR^; *Emx*1^Cre/+^) and control (*Htr1a*^*cR/cR*^; *Emx*1^+/+^) mice was measured. To be able to directly compare behavioral results from these mice with those of constitutive *Htr1a* knockout mice (*Htr1a*^*KO*^; Ramboz et al., 1998) *Htr1a* knockout littermates with and without the *Emx*1^Cre^ allele (*Htr1a*^KO/KO^; *Emx*1^Cre/+^ and *Htr1a*^KO/KO^; *Emx*1^+/+^) were included as controls. A significant effect of genotype was found on time in the open arms [Figure 5B; ANOVA, main effect of genotype: *F*_(3, 108)_ = 5.284, *P* = 0.0019] and number of head dips [Figure 5C; ANOVA, main effect of genotype: *F*_(3, 108)_ = 10.30, *P* < 0.0001] in the elevated plus maze. Cortex-specific rescue animals (*Htr1a*^cR/cR^; *Emx*1^Cre/+^) spent more time and performed more head dips, respectively, than their Cre-negative (*Htr1a*^cR/cR^; *Emx*1^+/+^) littermates. No genotype effect on total locomotion [Figure 5A; ANOVA, main effect of genotype: *F*_(3, 108)_ = 0.05335, *P* = 0.9837] was detected. Importantly, no significant effect of the Cre transgene alone on behavior (*Htr1a*^KO/KO^; *Emx*1^Cre/+^ vs. *Htr1a*^KO/KO^; *Emx*1^+/+^, *P* > 0.05 for all parameters analyzed) was detected. Moreover, a significant genotype effect was seen on distance traveled in the light compartment [ Figure 5D; ANOVA, main effect of genotype: *F*_(3, 86)_ = 5.629, *P* = 0.0014] as well as number of transitions between dark and light compartments [Figure 5F; ANOVA, main effect of genotype: *F*_(3, 86)_ = 3.504, *P* = 0.0188] in the dark/light box test. No genotype effect was observed on the time spend in the light compartment [Figure 5E; ANOVA, main effect of genotype: *F*_(3, 86)_ = 2.170, *P* = 0.0975]. No significant difference between *Htr1a* knockout animals with or without the Cre transgene was detected, arguing against an effect of this transgene on anxiety behavior under our testing conditions. Also no behavioral difference between *Htr1a*^cR/cR^ animals and constitutive knockout littermates (*Htr1a*^*KO/KO*^) was observed, suggesting that the relatively small difference in Htr1a expression between these lines was not sufficient to affect the behavioral phenotypes we scored. Moreover, a significant effect of genotype on total locomotion and a trend for decreased anxiety (increased time spent in center and % distance in center) was observed in cortex-specific rescue animals (*Htr1a*^cR/cR^; *Emx*1^Cre/+^) in the open field test (Supplementary Figure S1). Because it was not possible to include more than two alleles of *Htr1a* in the breeding, we were not able to directly compare the behavior of cortex-specific rescue mice to wild-type littermates. However, behavioral testing of wild-type and constitutive *Htr1a* knockout littermates deriving from a separate breeding confirmed the anxiety phenotype of the knockout mice (Ramboz et al., 1998; Bonasera and Tecott, 2000; Ase et al., 2002; Toth, 2003; Piszczek et al., 2013) under the breeding and testing conditions used in this study (Supplementary Figure S2). Finally, no genotype effect was seen on time spent immobile in the tail suspension test [Figure 6; ANOVA, main effect of genotype: *F*_(3, 108)_ = 0.8438, *P* = 0.4728]. Together these data demonstrate that increased expression of Htr1a in cortical structures is sufficient to induce reduced anxiety behavior.

## Discussion

Here we showed that selectively increasing Htr1a expression in cortical principal neurons using a novel Cre-inducible rescue allele of *Htr1a* is sufficient to decrease anxiety behavior in mice. The results presented in this work support our previous findings arguing for a role of forebrain Htr1a receptors in the modulation of anxiety (Gross et al., 2002). This, taken together with data showing that genetic suppression of Htr1a expression in serotonin neurons, but not forebrain is sufficient to increase anxiety (Richardson-Jones et al., 2011; Donaldson et al., 2014) and that selective rescue of the receptor in serotonin neurons does not rescue the knockout phenotype (Piszczek et al., 2013), argues for an interaction between forebrain and non-forebrain receptors in regulating this phenotype (Piszczek et al., 2013).

Several features of our Cre-inducible Htr1a allele contributed to our results. First, Cre-induced Htr1a expression is under the control of the endogenous *Htr1a* promoter and was restricted to the endogenous expression pattern (Figure 2) and cell-types (Figure 3). This is an improvement on our previous *Htr1a* genetic rescue strategy (Gross et al., 2002) that relied on the *Camk2a* promoter and was associated with both over-expression and ectopic expression of Htr1a. Second, *Htr1a*^cR^ mice exhibited low, but significant levels of Htr1a expression (Figures 2, 4) that allowed us to examine the effect of raising Htr1a protein levels, rather than restoring it to mice with no endogenous receptor expression. Arguably, this is a more subtle manipulation of Htr1a receptor levels that may have facilitated our ability to elicit changes in anxiety behavior that depend, for example, on autoreceptor expression. Interestingly, we found that *Htr1a*^cR/cR^ mice were indistinguishable from knockout mice in our behavioral tests. Although the *Htr1a* knockout allele is dosage sensitive (Ramboz et al., 1998) and heterozygous mice show intermediate levels of protein expression as well as behavior. Our findings suggest that the small difference in gene expression between *Htr1a*^cR/cR^ and *Htr1a*^KO/KO^ mice was not sufficient to affect the behavioral phenotypes we measured. Third, the Cre-dependence of our Htr1a allele allowed us to use the *Emx1*-Cre transgene to drive rescue of Htr1a selectively in principal cortical neurons (Figure 4). Htr1a has been shown to be expressed not only in glutamatergic principal cells in cortex, hippocampus, and amygdala (Aznar et al., 2003; Santana et al., 2004; Palchaudhuri and Flügge, 2005; De Almeida and Mengod, 2008; Marvin et al., 2010), but also in GABAergic interneurons in these structures (Aznar et al., 2003; Santana et al., 2004; De Almeida and Mengod, 2008). Others have shown, that decreasing the expression on the excitatory principal neurons, but not interneurons, does not affect anxiety behavior, but leads to depression-like behavior (Richardson-Jones et al., 2011). Our approach allowed us to define more precisely the cell-types involved in anxiety modulation by Htr1a, excluding a role for non-cortical structures as well as cortical interneurons, showing that increasing the expression on the principal cells, but not interneurons, decreases anxiety levels without affecting depression-like behavior. Taken together these data suggest different and/or compensatory role of the two subpopulations of Htr1a heteroreceptor on both anxiety and depression-like behaviors.

Our failure to observe a change in depression-related behavior in the tail-suspension test was likely due to the absence of a phenotype in our knockout mice under these test conditions. Several other studies have reported decreased immobility in the forced swim and tail suspension test in *Htr1a* knockout mice (Ramboz et al., 1998; Mayorga et al., 2001). It is unclear why we did not replicate this finding in our mice, but differences in strain or testing procedure may have contributed. Despite earlier reports to the contrary (Cao and Li, 2002), we did not see any alterations in anxiety behavior associated with the *Emx1*-Cre allele on the knockout background (Figure 5). This argues against a contribution of the Cre driver line (via *Emx1* heterozygosity or linked-polymorphisms) in the decreased anxiety observed in the cortical rescue mice.

In conclusion, our data support an anxiolytic role for Htr1a expressed on neocortical (including cortical-related nuclei such as basolateral amygdala) and hippocampal (archicortex) principal cells, consistent with a role for cortical structures in modulating anxiety. Earlier data showed that the Htr1a knockout phenotype depends on Htr1a expression during the early postnatal period when it is able to exert an influence on dendritic branching by modulating actin-dependent growth cone dynamics in CA1 hippocampal pyramidal neurons (Ferreira et al., 2010). Moreover, our previous work suggested that the anxiety phenotype of *Htr1a* knockout mice has a cognitive component that is likely to be mediated by deficits in cortical processing of threat information (Klemenhagen et al., 2006; Tsetsenis et al., 2007; Lo Iacono and Gross, 2008). Thus, increase of Htr1a expression in principal hippocampal neurons may be involved in the reduction of anxiety levels in our mice, as supported by data demonstrating a function of the hippocampus in encoding the probability of conditioned stimulus association (Múnera et al., 2000) and the evaluation of ambiguous threat cues (Tsetsenis et al., 2007). Our findings demonstrate a role for Htr1a heteroreceptors in the developmental programming of cortical circuits in such a way so as to positively bias the evaluation of threats. Negative threat bias has been described as a major component of neuroticism as well as mood disorders (Roy et al., 2008; Armstrong and Olatunji, 2012; Maalouf et al., 2012; Hommer et al., 2014) and defining genetic modulators of the relevant brain regions in mice may help to better define anxiety-related mental traits in humans with the long-term goal of identifying targeted therapeutic interventions.

### Conflict of interest statement

The authors declare that the research was conducted in the absence of any commercial or financial relationships that could be construed as a potential conflict of interest.
