# Supplementary material for: Modulation of anxiety by cortical serotonin 1A receptors
Source: Front Behav Neurosci. 2015 Feb 24;9:48. doi: 10.3389/fnbeh.2015.00048 (PMC4338812; doi:10.3389/fnbeh.2015.00048)
Supplement: Supplementary file 1 [file DataSheet1.DOCX]

***Supplementary Material***

**Modulation of anxiety by cortical serotonin 1A receptors**

**Lukasz Piszczek^1^, Agnieszka Piszczek^1^, Joanna Kuczmanska^1^, Enrica Audero^1^, Cornelius T. Gross^1,*^**

^1^ Mouse Biology Unit, European Molecular Biology Laboratory (EMBL), Via Ramarini 32, 00015 Monterotondo, Italy

*** Correspondence:** Cornelius T. Gross, Mouse Biology Unit, European Molecular Biology Laboratory (EMBL), Via Ramarini 32, 00015 Monterotondo, Italy.

**Supplementary Figures**

Additionally the adult cortical rescue (*Htr1a*^cR/cR^;*Emx1*^Cre/+^) and control mice were tested in the open field arena (**Supplementary Figure 1**). A significant effect of genotype was found on total locomotion (ANOVA, main effect of genotype: F_(3,108)_ = 3.125, P = 0.0285), but not on time in center (ANOVA, main effect of genotype: F_(3,108)_ = 1.937, P = 0.1279) nor % distance in center (ANOVA, main effect of genotype: F_(3,108)_ = 2.171, P = 0.0956). However, cortical rescue animals showed a trend for increased time in center as well as % center distance as compared to their non-Cre littermates. Similarly to elevated plus maze and dark/light box, no significant difference between *Htr1a* knockout animals with or without the Cre transgene was observed arguing against an effect of this transgene on anxiety behavior under our testing conditions.


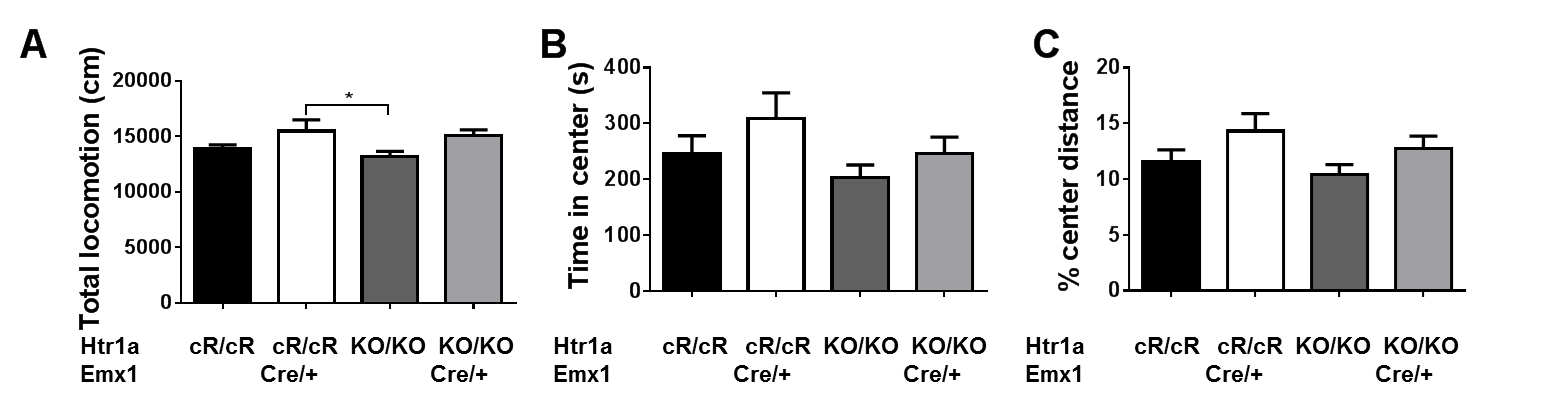


Supplementary Figure 1. Trend for decreased anxiety in cortex rescue animals in open field test. Testing of control (*Htr1a*^cR/cR^), cortical rescue (*Htr1a*^cR/cR^;*Emx1*^Cre/+^), knockout (*Htr1a*^KO/KO^), and knockout mice carrying the *Emx1*-Cre allele (*Htr1a*^KO/KO^;*Emx1*^Cre/+^) in the open field tests. A significant effect of genotype was seen on (A) total locomotion. Furthermore, trend for increased time in center (B) and % center distance (C) was seen in cortical rescue mice as compared to their non-Cre littermates (*Htr1a*^cR/cR^, N = 25; *Htr1a*^cR/cR^;*Emx1*^Cre/+^, N = 25; *Htr1a*^KO/KO^, N = 36; *Htr1a*^KO/KO^;*Emx1*^Cre/+^, N = 26).

In a parallel cohort of animals coming from a separate breeding the effect of the constitutive knockout of the *Htr1a* was tested in the same behavioral paradigms as described for the Htr1a-cR line. In the elevated plus maze (**Supplementary Figure 2 A-C**) a significant decrease in total locomotion (one-tailed t-test, P = 0.0076) and number of head dips (one-tailed t-test, P = 0.0241) was observed, with a trend for decreased % time spent in open arms (one-tailed t-test, P = 0.1011) in the knockout (*Htr1a*^KO/KO^) animals as compared to wild-type (*Htr1a*^+/+^) littermates. In the dark/light box test (**Supplementary Figure 2 D-F**) a significant decrease in distance traveled (one-tailed t-test, P = 0.0282) and time spent in light compartment (one-tailed t-test, P = 0.0231) as well as number of entries to light compartment (one-tailed t-test, P = 0.0014) was observed in knockout (*Htr1a*^KO/KO^) animals when compared to wild-type (*Htr1a*^+/+^) littermates. Finally, in the open field test (**Supplementary Figure 2 G-I**) a significant decrease in time spent in center (one-tailed t-test, P = 0.0025) as well as % distance travelled in the center area (one-tailed t-test, P = 0.0002) without a significant change in total locomotion (one-tailed t-test, P = 0.1423) was observed in the knockout (*Htr1a*^KO/KO^) animals when compared to wild-type (*Htr1a*^+/+^) littermates. These data confirm that constitutive *Htr1a* knockout animals exhibit significant increases in anxiety-related measures under the testing conditions as used for the Htr1a-cR line.

Supplementary Figure 2. Increased anxiety in constitutive *Htr1a* knockout mice. Testing of control wild-type (WT, *Htr1a*^+/+^) and knockout (KO, *Htr1a*^KO/KO^) littermates in the elevated plus maze (A-C), dark/light (D-F) and open field (G-I) tests. A significant decrease in (A) total locomotion and (C) number of head dips in the elevated plus maze (*Htr1a*^+/+^, N = 18; *Htr1a*^KO/KO^, N = 32). Similarly, a significant decrease in (D) distance and (E) time spent, as well as (F) entries in light compartment was seen in knockout animals (*Htr1a*^+/+^, N = 19; *Htr1a*^KO/KO^, N = 24). Finally, a significant decrease in (H) time and (I) distance travelled in center and no change in (G) total locomotion was seen in knockout animals in the open field test (*Htr1a*^+/+^, N = 26; *Htr1a*^KO/KO^, N = 37).
